# LncRNA KCNQ1OT1 predicts further cerebral events in patients with transient ischemic attack

**DOI:** 10.3389/fphar.2022.961190

**Published:** 2022-10-07

**Authors:** Shijia Yu, Jing An, Ran Sun, Juan Feng, Mingjun Yu

**Affiliations:** ^1^ Department of Neurology, Shengjing Hospital of China Medical University, Shenyang, China; ^2^ Department of Neurosurgery, Shengjing Hospital of China Medical University, Shenyang, China

**Keywords:** lncRNAs, transient ischemic attack, KCNQ1OT1, hs-CRP, inflammation, cerebral ischemic stroke

## Abstract

Transient ischemic attack (TIA) poses a great threat of cerebrovascular diseases to a large number of patients, despite its reversible neurological dysfunction. Long non-coding RNAs (lncRNAs) have been proven to play critical roles in the pathophysiological development of cerebrovascular events. Exploring the function of lncRNAs in modulating TIA prognosis would help to develop individualized therapeutics. A total of 231 participants with the first onset of TIA were recruited in the study, including 65 subsequent stroke patients. The expression of lncRNA potassium voltage-gated channel subfamily Q member 1 opposite strand 1 (KCNQ1OT1) was upregulated in patients with recurrent ischemic events after TIA. Additionally, KCNQ1OT1 could be regarded as an independent predictor for subsequent ischemic stroke. The optimal diagnostic value was determined at 1.29 with a sensitivity of 63% and a specificity of 72%. Fewer patients would survive from further ischemic stroke with their KCNQ1OT1 level over 1.29. Furthermore, the expression of KCNQ1OT1 was elevated with a growing serum high-sensitivity C-reactive protein (hs-CRP) level. KCNQ1OT1 might be involved in the regulation of early inflammatory response during recurrence of TIA.

## Introduction

Transient ischemic attack (TIA) is one of the most common alerting events for ischemic cerebral vascular attacks, with the sudden onset of neurological disorders such as paralysis, paresthesia, and speech, visual, or hearing impairments. These symptoms were temporary and alleviated in 24 h without any sequelae. However, these patients are at a high risk of further ischemic events after TIA. According to a systemic review of several follow-up studies, the approximate proportion of further stroke in patients with a first episode of TIA varied from approximately 8% to 12% in 7 days; however, it varied from 10% to 22% in 90 days (Giles and Rothwell, 2007). Risk levels of TIA which were measured by the ABCD2 score scale exerted pivotal roles in predicting subsequent ischemic stroke of TIA (Giles and Rothwell, 2007; Perry et al., 2021), whereas patients were usually scored depending on their medical histories and characteristic clinical manifestations. In addition to this, we wondered whether any molecular biomarkers could help to evaluate the latent possibility for recurrent cerebral ischemia after TIA.

According to previous studies, inflammation could exacerbate cerebral ischemic stroke by inducing atherosclerosis, facilitating instability of plaque, as well as overactivating platelets and coagulation (Esenwa and Elkind, 2016; Patel et al., 2018). The expression of C-reactive protein (CRP) was upregulated within 24 h after acute stroke and will sustain at a high level for nearly 6 months (Arenillas et al., 2003). Both clinical evidence and laboratory evidence suggested that anti-inflammatory therapies effectively decreased vascular damages and suppressed cerebral ischemic stroke (Kelly et al., 2018). Moreover, a high level of residual inflammatory risk (RIR) was proven to imply patients’ poor prognosis of acute cerebral ischemic stroke (Li et al., 2021a), while high-sensitivity C-reactive protein (hs-CRP), which represented early inflammatory response, was addressed as a decisive factor of RIR (Ridker et al., 2017). Low-level inflammation reflected by hs-CRP indicated recurrent ischemic events independently (Mengozzi et al., 2020; Coveney et al., 2021).

Potassium voltage-gated channel subfamily Q member 1 opposite strand 1 (KCNQ1OT1) is defined as a long non-coding RNA with more than 200 nucleotides. It was the overlapping transcript 1 located on the opposite strand of KCNQ1. KCNQ1OT1 influenced multiple target gene expression and function through epigenetic modifications (Pandey et al., 2008). Previously, we verified that KCNQ1OT1 could accelerate autophagy of neurons, thus exacerbating ischemia and reperfusion (I/R) injury in acute cerebrovascular diseases (Yu et al., 2019). Evidence demonstrated that KCNQ1OT1 activated inflammatory response and promoted apoptosis of microvascular endothelial cells in acute myocardial infarction (Wang et al., 2019; Liao et al., 2020). Moreover, KCNQ1OT1 could aggravate atherosclerosis *via* regulation of lipid metabolism (Yu et al., 2020). Nevertheless, little has been known about the effects of KCNQ1OT1 on subsequent ischemic stroke after TIA.

In our study, the mounting expression of KCNQ1OT1 was detected in plasma from TIA patients. Moreover, early inflammation illustrated by the serum hs-CRP level was positively associated with KCNQ1OT1 expression. Our findings will provide not only a novel biomarker for the risk of recurrence but also the latent therapeutic target in patients who suffered TIA.

## Materials and methods

### Ethics statement

This study was a clinical observational cohort study. The Institutional Review Board of Shengjing Hospital of China Medical University had approved our study (IRB number, 2017PS161K). Written informed consent was provided by all the participants or their legal representatives.

### Patient collection

A total of 231 individuals hospitalized at Shengjing Hospital from January 2017 to May 2018 were recruited into our study. All of them had experienced their first sudden onset of TIA events within 24 h and were diagnosed by two specialists in stroke treatment according to the diagnostic criteria (Simmons et al., 2012; Easton and Johnston, 2022). These patients complained transient neurological dysfunction, including weakness of limbs, paresthesia, and aphasia. These symptoms were relieved in 1 day without any treatment. Computed tomography (CT) scans were conducted to exclude other brain diseases such as cerebral hemorrhage or tumor. Patients with poor health condition and with chronic, infectious, or systemic diseases, including cardiovascular disease, diabetes, tumor, liver, kidney, or other cerebral diseases, were excluded from our study. In addition, individuals with atrial fibrillation or mural thrombus detected by echocardiogram were also excluded. Venous blood was collected for laboratory tests when patients were hospitalized. Basic information was collected and analyzed. Carotid atherosclerosis was detected by ultrasonography.

### Follow-ups

To observe recurrent ischemic events in 3 months, our research associates followed up all the individuals in-person or online at 7, 14, 30, and 90 days after the first onset of TIA. All the participants followed doctors’ directions for secondary prevention strictly. During the investigation, the primary outcome was set as the onset of recurrence of ischemic stroke, while the secondary outcome was all-cause death. Among all the participants, there were 65 patients suffering subsequent stroke.

### Detection of plasma lncRNA KCNQ1OT1

Blood samples were centrifuged to obtain the supernatant plasma at the first onset of TIA. The TRIzol reagent (Life Technologies Corporation, Carlsbad, CA, United States) was utilized to dissociate the total RNAs from patients’ plasma. Then, the plasma KCNQ1OT1 level was detected *via* real-time quantitative polymerase chain reaction (qPCR) using the One-Step SYBR Primer Script RT-PCR Kit (Takara Bio, Inc., Japan). The total volume of the reaction system is 20 μl. Melt curves were analyzed to determine the specificity of the amplified products. Primer sequences for KCNQ1OT1 detection were designed as follows: forward 5′-TGC​AGA​AGA​CAG​GAC​ACT​GG-3′ and reverse 5′-CTT​TGG​TGG​GAA​AGG​ACA​GA-3’. GAPDH was regarded as the endogenous reference with the following sequences of primers: forward 5′- TGC​ACC​ACC​AAC​TGC​TTA​GC-3′ and reverse 5′-GGC​ATG​CAC​TGT​GGT​CAT​GAG-3’.

### Assessment of early inflammatory response

The level of hs-CRP was tested to evaluate the early inflammatory response in all cases. Immunoturbidimetric analysis was conducted according to the manufacturer’s instructions (Beckman Coulter Inc., Brea, CA, United States). The IMMAGE 800 Immunochemistry System (Beckman Coulter Inc., Brea, CA, United States) was utilized for the analysis.

### Further stroke risk prediction

The risk for further stroke after TIA was determined by two experienced neurologists using the ABCD2 score scale based on demographic characteristics and clinical manifestations (Wardlaw et al., 2015). Patients who scored 0–3 were considered to be at the low-risk level, while those who scored 4–5 and 6–7 were considered to be at the moderate-risk level and at the high-risk level, respectively.

### Statistical analysis

Measurement data were presented as mean ± SD. Student’s t-test or two-way ANOVA was applied for parametric comparison between data with normal distribution. Non-normal distributed data were analyzed by the Mann–Whitney *U*-test. The percentage of measurement data was calculated and compared using the chi-squared test. The Kruskal–Wallis test was used for the nonparametric comparison between multiple groups. Logistic regression analysis was conducted to determine latent independent risk factors for subsequent ischemic stroke with odd ratios (ORs) and 95% confidence intervals (CIs). The relationship between the expression of KCNQ1OT1 and ABCD2 score or hs-CRP was evaluated through Spearman analysis. The receiver operating characteristic (ROC) curve was applied to calculate the predictive value of KCNQ1OT1 in further events after TIA. Kaplan–Meier analysis was applied to draw the survival curve in the follow-up study. All the statistics were performed using SPSS 23.0 (IBM Corp., Armonk, NY, United States). *p* < 0.05 was considered statistically significant.

## Results

### KCNQ1OT1 is an independent risk factor for subsequent ischemic stroke

**FIGURE 1:**
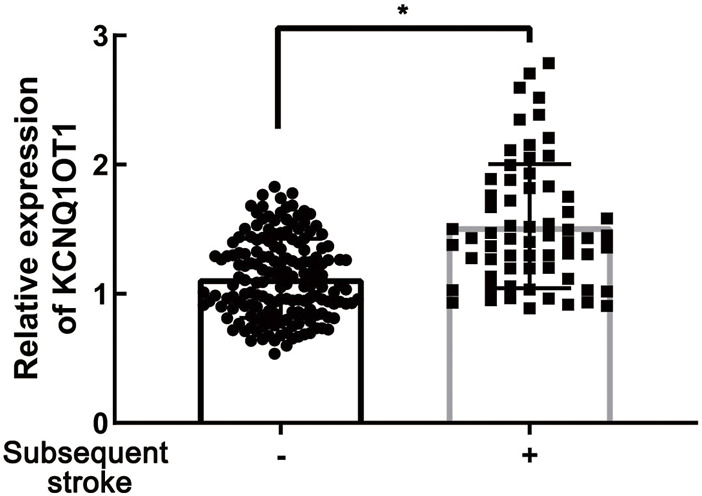
KCNQ1OT1 was prominently upregulated in TIA patients with subsequent ischemic stroke events, *p < 0.05.

To determine the role of KCNQ1OT1 after TIA, we studied peripheral plasma samples from 231 individuals, including 166 patients without further neurological dysfunction after TIA and 65 patients who suffered subsequent ischemic stroke. Demographic characteristics are listed in Table 1. Clinical assessments of BMI and alcohol assumption, *etc.*, were performed as previously described (Yu et al., 2020). There were differences with risk factors such as hypertension, diabetes, and carotid stenosis between these two groups (*p* < 0.05). The relative expression of KCNQ1OT1 was detected by qPCR. It showed that the KCNQ1OT1 level was prominently upregulated in the subsequent stroke- positive group (*p* < 0.05; Figure 1). Moreover, logistic regression analysis was performed to elucidate that KCNQ1OT1 was an independent risk factor for further ischemic events in TIA patients (*p* < 0.001, OR 21.591, 95% CI 5.884–79.220; Table 2).

**TABLE 1:** Baseline characteristics of the subjects.

	Total *n* = 231	Subsequent stroke (−) *n* = 166	Subsequent stroke (+) *n* = 65	*p*-value
Age (years)	62.98 ± 7.01	62.43 ± 6.40	64.40 ± 8.26	0.055
Male, *n* (%)	113 (48.9)	76 (45.8)	37 (56.9)	0.128
BMI (kg/m^2^)	26.09 ± 5.43	26.23 ± 5.48	25.74 ± 5.34	0.541
Waist circumference (cm)	89.29 ± 7.41	88.79 ± 7.66	90.57 ± 6.61	0.102
Risk factors, *n* (%)				
Current smoking	71 (30.7)	47 (28.3)	24 (36.9)	0.202
Alcohol consumption	53 (22.9)	35 (21.1)	18 (27.7)	0.283
Hypertension	151 (65.4)	102 (61.4)	49 (75.4)	0.045*
Diabetes	124 (53.7)	82 (49.4)	42 (64.2)	0.037*
Carotid stenosis	57 (24.7)	35 (21.1)	22 (33.8)	0.043*
Laboratory tests				
Total cholesterol (mmol/L)	4.97 ± 1.27	4.93 ± 1.26	5.06 ± 1.31	0.513
Triglyceride (mmol/L)	1.47 ± 0.61	1.45 ± 0.55	1.53 ± 0.74	0.371
LDL-C (mmol/L)	2.77 ± 0.69	2.74 ± 0.68	2.85 ± 0.71	0.287
HDL-C (mmol/L)	1.08 ± 0.37	1.07 ± 0.39	1.12 ± 0.33	0.376
ABCD2 score, *n* (%)				0.000*
Low risk, 0-3	73 (31.6)	62 (37.3)	11 (16.9)	
Moderate risk, 4-5	97 (41.9)	72 (43.4)	25 (38.5)	
High risk, 6-7	61 (26.5)	32 (19.3)	29 (44.6)	

BMI, body mass index; LDL, low-density lipoprotein; HDL, high-density lipoprotein. *p < 0.05 was considered statistically significant.

**TABLE 2:** Logistic regression analysis of independent risk factors.

	OR	95% CI for OR		*p*-value
		Lower	Upper	
Hypertension	1.395	0.444	4.387	0.569
Diabetes	0.665	0.210	2.110	0.489
Carotid stenosis	0.562	0.231	1.365	0.203
KCNQ1OT1	21.591	5.884	79.220	0.000*

*p < 0.001 was considered statistically significant.

### KCNQ1OT1 is positively associated with the risk levels of transient ischemic attack

**FIGURE 2:**
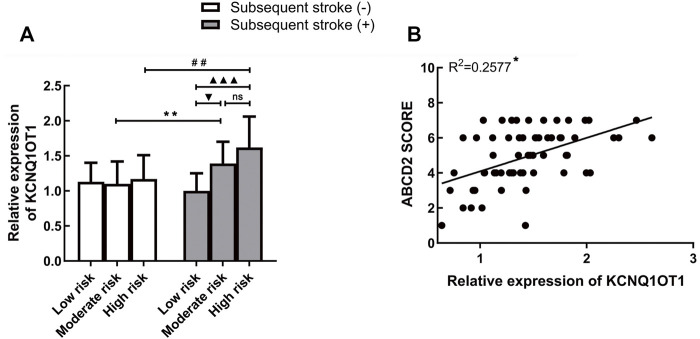
KCNQ1OT1 was associated with risk levels of TIA. **(A)** KCNQ1OT1 was increased significantly in subsequent stroke-positive cases at moderate or high risk. *p < 0.05 vs. subsequent stroke (+) group at moderate risk^. #^ *p* < 0.05 vs. subsequent stroke (+) group at high risk. **(B)** Expression of KCNQ1OT1 was positively associated with the ABCD2 score in TIA patients with subsequent stroke events (*n* = 65, *p < 0.05).

Risk stratification of stroke in TIA was decided on the basis of the ABCD2 scoring system (low risk: 0–3, moderate risk: 4–5, and high risk: 6–7). As shown in Table 1, there are differences in the proportion of the three risk levels from two groups. Therefore, we wondered whether the expression of KCNQ1OT1 was changed in patients at different risk levels. Three subgroups were framed out according to their stroke risks from low to high level. In the moderate/high-risk subgroups, KCNQ1OT1 expression was significantly upregulated in patients who suffered further ischemic events. For those who had subsequent stroke after TIA, the plasma KCNQ1OT1 level was increased strikingly in moderate/high-risk subgroups (Figure 2A). In addition, the ABCD2 score elevated along with the increased expression of KCNQ1OT1 in TIA patients who suffered recurrent stroke (*R* ^*2*^ = 0.2577, *p* < 0.05; Figure 2B).

### KCNQ1OT1 predicts further ischemic events after transient ischemic attack

**FIGURE 3:**
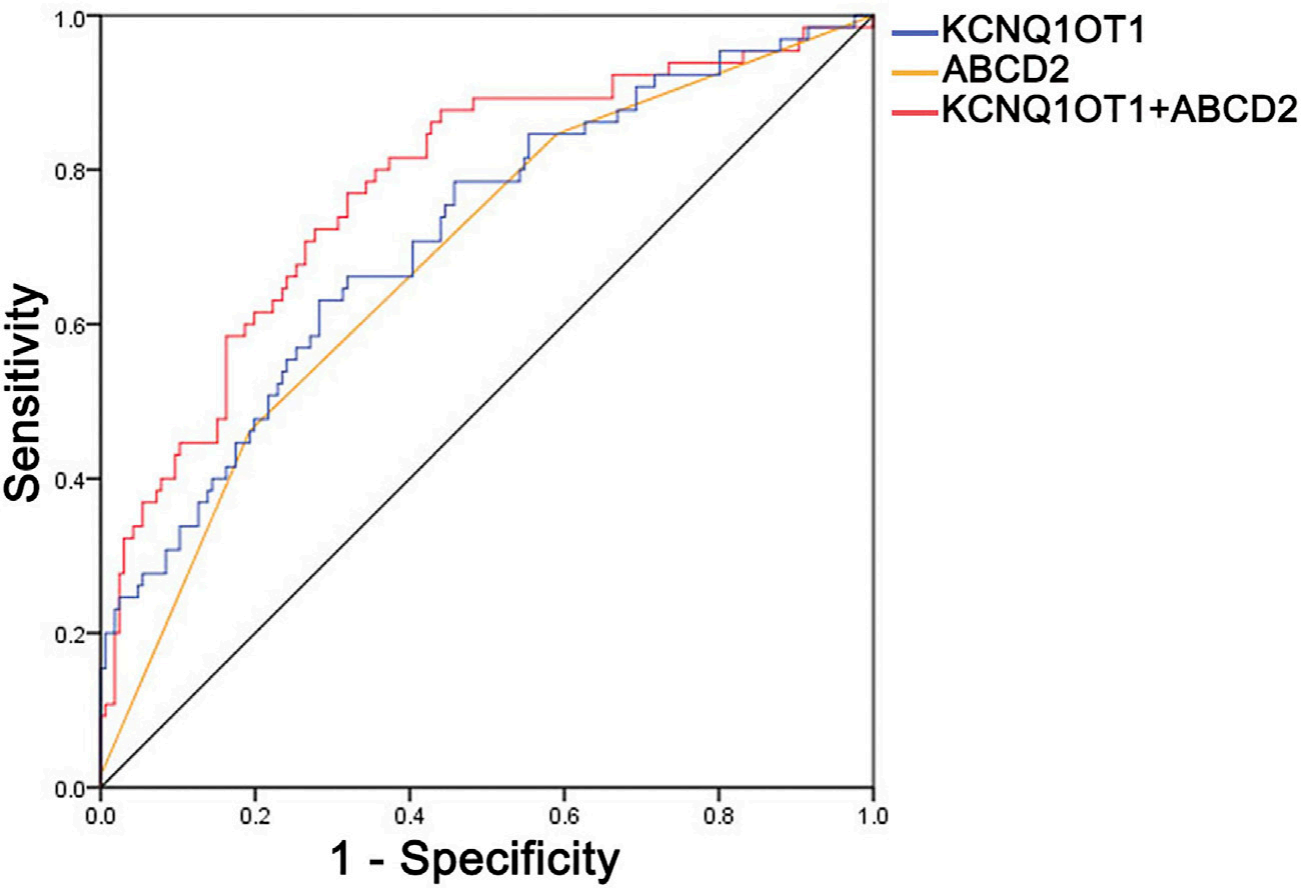
Receiver operating characteristic curve (ROC) of KCNQ1OT1, ABCD2-based risk level, and their combined application. The values of the area under the curve were 0.714, 0.672, and 0.778, correspondingly, with 63% sensitivity and 72% specificity of KCNQ1OT1 and 67.7% sensitivity and 76.5% specificity of KCNQ1OT1 combined with the ABCD2-based risk level.

To depict more clinical significance of KCNQ1OT1 in TIA, a ROC curve was drawn according to the relative expression of KCNQ1OT1 in plasma, ABCD2-based risk levels, and combination of them (Figure 3). The area under the ROC curve (AUC) was 0.714, 0.672, and 0.778, correspondingly. KCNQ1OT1 was considered the predictive factor for further events after TIA, with a sensitivity of 63% and specificity of 72%. An optimal diagnostic point for the relative expression of KCNQ1OT1 was determined at 1.29 *via* the Youden Index analysis. Combined with ABCD2 score scale-based risk levels, sensitivity and specificity of predictive factors were increased up to 67.7% and 76.5%, correspondingly. These results suggested that KCNQ1OT1 could predict further ischemic stroke events and improve the efficacy of evaluating risk levels of TIA patients by ABCD2 criteria.

### KCNQ1OT1 expression level over 1.29 indicated a lower survival rate from further ischemic events

**FIGURE 4:**
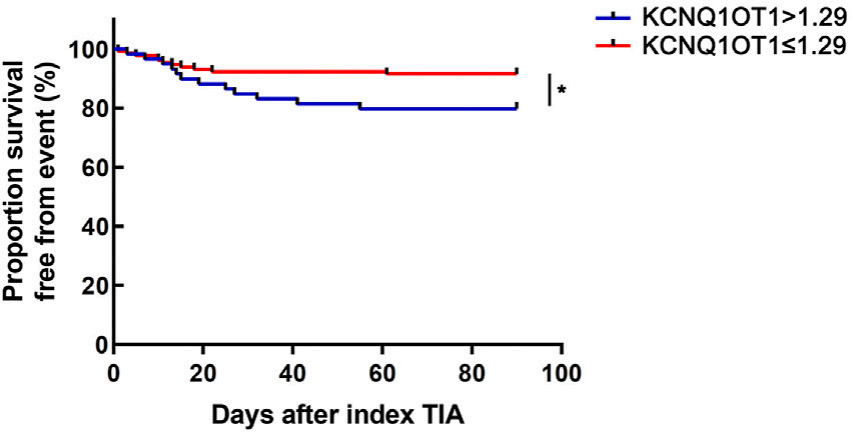
KCNQ1OT1 > 1.29 indicated poor outcomes in the following 90 days, *p < 0.05.

According to the optimal diagnostic point, the expression level of KCNQ1OT1 over 1.29 was determined as an independent predictor for subsequent stroke events (*p* < 0.05, OR 6.142, and 95% CI 2.723–13.857; Table 3). Furthermore, the survival curve was established using the Kaplan–Meier analysis during a 90-day follow-up. It was clearly demonstrated that fewer patients could survive from the subsequent ischemic events when their plasma KCNQ1OT1 level was over 1.29 (*p* < 0.001; Figure 4).

**TABLE 3:** Expression of KCNQ1OT1 > 1.29 predicts further ischemic stroke.

	OR	95% CI for OR		*p*-value
		Lower	Upper	
Hypertension	2.219	0.748	6.583	0.151
Diabetes	0.783	0.270	2.272	0.653
Carotid stenosis	0.444	0.172	1.143	0.092
KCNQ1OT1 > 1.29	6.142	2.723	13.857	0.000*

*p < 0.001 was considered statistically significant.

### KCNQ1OT1 is related to hs-CRP in patients with subsequent ischemic stroke

**FIGURE 5:**
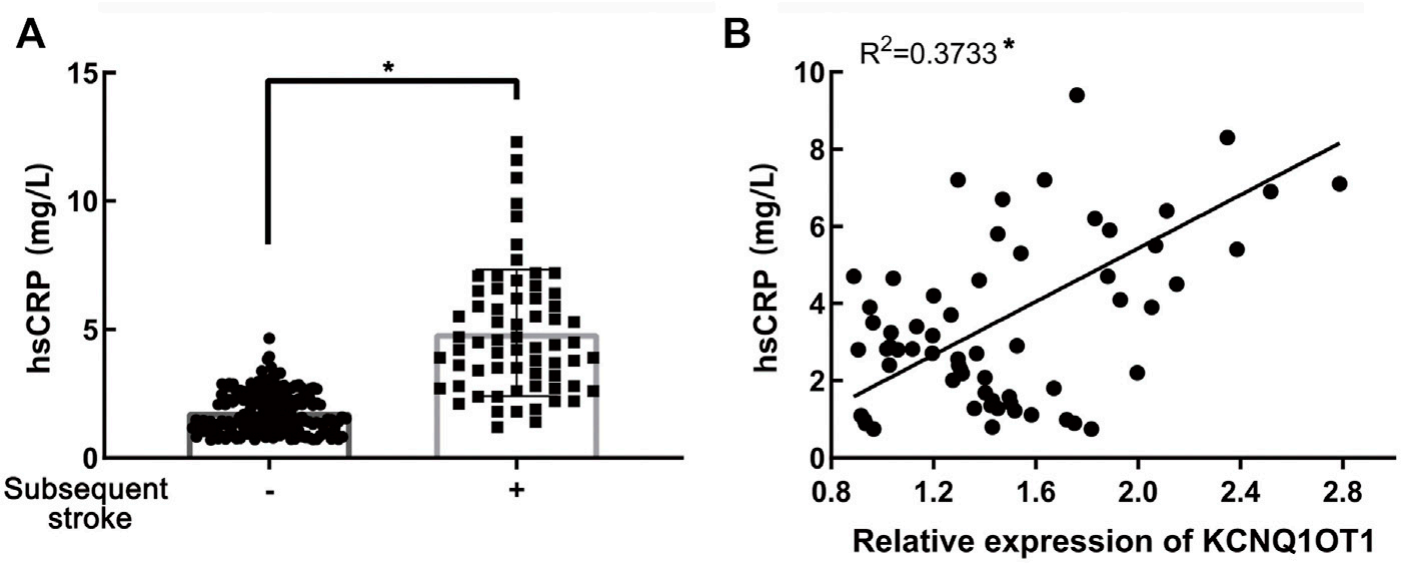
Relationship of KCNQ1OT1 and hs-CRP in further ischemic events. **(A)** hs-CRP level was elevated in patients with further ischemic stroke, *p < 0.05. **(B)** hs-CRP level was positively related to the expression of KCNQ1OT1 in the subsequent stroke-positive group (*n* = 65, *p < 0.05).

Since early inflammatory response reflected by hs-CRP was proven to play pivotal roles in ischemic stroke following TIA (Mengozzi et al., 2020), we wondered whether there was any association between the expression of KCNQ1OT1 and hs-CRP in plasma. Enzyme-linked immunosorbent assays (ELISA) implicated that the hs-CRP level was higher in the subsequent stroke-positive group (*p* < 0.05; Figure 5A). Further study revealed that hs-CRP was increased with the increase in the expression of KCNQ1OT1 in patients who suffered further ischemic events after TIA (*R* ^*2*^ = 0.3733, *p* < 0.05; Figure 5B). This result implied that KCNQ1OT1 might participate in the early inflammatory response in further stroke after TIA.

## Discussion

In this study, we proposed that the expression of KCNQ1OT1 was upregulated in further ischemic events and positively associated with the stroke risk levels of TIA for the first time. Moreover, our study indicated that KCNQ1OT1 could be regarded as a predictive factor for subsequent stroke. With the expression level of KCNQ1OT1 over 1.29, the survival rate in 90 days after the first onset of TIA was decreased apparently. In addition, the early inflammatory response detected by hs-CRP was enhanced with an increasing KCNQ1OT1 level.

TIA has been considered the predominant risk factor for further stroke of unreversible neurological impairments. Studies reported that patients possessed 5.4%–14.2% possibility to develop into permanent neurological deficits after their first onset of TIA (Khanevski et al., 2019). Although neurologists strived to prohibit recurring TIA, there were still 25–30% of patients who suffered secondary stroke within 5 years after TIA (Valls et al., 2017). Currently, patients’ age, blood pressure, diabetes history, clinical manifestations, and duration of clinical symptoms are the most acknowledged factors that would influence further stroke after TIA (Wardlaw et al., 2015). Recent research revealed that patients whose systolic blood pressure was over 140 mmHg exposed a higher risk of recurring neurological dysfunction (de Havenon et al., 2021). Moreover, studies also discovered that further stroke was more likely to happen in TIA patients with intracranial atherosclerosis and aberrant inflammatory response (Luijendijk et al., 2011; Colas-Campas et al., 2020; Lindenholz et al., 2020). In the previous study, we have proven that overactivated autophagy and apoptosis of neurons could exacerbate cerebral ischemia–reperfusion injury and aggravated neurological impairments (Yu et al., 2020; Yu et al., 2021). In spite of no neurological deficits left, TIA was also caused by cerebral ischemia. Thus, we further studied the latent factors reflecting the development and prognosis of TIA.

Mounting evidence manifested that lncRNA KCNQ1OT1 participated in regulating cell progression and differentiation among various diseases such as cancer, fracture, and myocardial infarction (Feng et al., 2020; Wang et al., 2020; Li et al., 2021b; Hong et al., 2021). According to the previous study, KCNQ1OT1 was upregulated in the ischemic brain tissue, and its expression was growing with the patients’ severity of neurological impairments. Knockdown of KCNQ1OT1 could attenuate neural apoptosis induced by autophagy (Yu et al., 2019). In cerebral ischemia–reperfusion injury, silencing KCNQ1OT1 would attenuate endoplasmic reticulum stress *via* regulating the downstream miR-30b/GRP78 signaling cascade (Li et al., 2020). Moreover, KCNQ1OT1 aggravated fibrosis and pyroptosis in diabetic cardiomyopathy by targeting the miR-214-3p/caspase-1/TGF-β1/smad pathway (Yang et al., 2018). KCNQ1OT1 could competitively bind with miR-466 to mediate downstream Tead1 expression, promoting apoptosis of cardiomyocytes during acute myocardial infarction (Liao et al., 2020). Evidence also showed that the rising level of KCNQ1OT1 repressed cholesterol excretion and exacerbated lipid deposition in macrophages, which could facilitate the formation of atherosclerosis (Yu et al., 2020). However, there were few research studies about the roles of KCNQ1OT1 in TIA. In this study, we detected the upregulated level of KCNQ1OT1 and proposed KCNQ1OT1 as an independent risk factor in patients with further ischemic events after TIA. In addition, an optimal diagnostic value of KCNQ1OT1 expression as 1.29 was determined using the ROC curve analysis. Furthermore, we explored the underlying pathophysiological process associated with KCNQ1OT1 in TIA.

Inflammatory response was proven to be activated in acute cerebral cardiovascular diseases. In cerebral ischemia–reperfusion (I/R) injury, NLRP3 inflammasomes were accumulated in neurons and induced immune cell infiltration to destroy the integrity of the blood–brain barrier (Franke et al., 2021). In addition, microglial HDAC3 stimulated neuroinflammation and aggravated neurological impairments in I/R injury *via* the cGAS-STING pathway (Liao et al., 2020). Studies have reported that activated inflammation, which was reflected by the CRP level over 2 mg/L, would predict a high probability of recurrent cardiovascular diseases (McMurray et al., 2009; Blanco-Colio et al., 2021). In addition, inflammation could increase vulnerability of atherosclerotic plaques, which is the most common cause of recurrent ischemia (Badimon and Vilahur, 2014; Brinjikji et al., 2016). Inflammatory cytokines including IL-1β, IL-8, and hs-CRP were considered to predict recurrent ischemic stroke independently (Coveney et al., 2021). However, other researchers found that IL-6 and S-100B were decreased in the further events of TIA (Colas-Campas et al., 2020). Anti-inflammatory therapy with salvianolic acid D could impede the activation of the NF-κB pathway mediated by HMGB1 translocation in cerebral I/R injury (Zhang et al., 2020). Arginine acted as a neuroprotector to alleviate neurological impairments *via* inhibiting HIF-1α/LDHA-induced inflammation in microglia (Chen et al., 2020). Moreover, exosomes with conjugated curcumin could target the suppression of cerebral inflammatory response, so as to reduce neural apoptosis (Tian et al., 2018). As previously reported, KCNQ1OT1 could aggravate inflammatory response in myocardial infarction *via* the Notch pathway (Wang et al., 2019). Oxidative stress following myocardial infarction could be accelerated *via* the KCNQ1OT1/miR-130a-3p/ZNF791 axis (Xin et al., 2022). In addition, knockdown of KCNQ1OT1 would reduce the activation of NLRP3 inflammasome in acute kidney injury (Wang et al., 2021). Recent studies put forward that a high level of hs-CRP reflecting the early inflammation was an independent predictor for ischemic stroke after TIA or minor stroke (Mengozzi et al., 2020). Thus, we wondered whether the expression of KCNQ1OT1 was related to hs-CRP in further ischemic events after TIA. In our study, hs-CRP expression was found to be elevated up to 2.25 folds in patients with subsequent stroke, which was in agreement with the previous findings. Furthermore, we explored the latent association between upregulated KCNQ1OT1 expression and activated early inflammation reflected by hs-CRP in patients who had suffered further ischemic events of TIA. It was confirmed that the level of KCNQ1OT1 increased with the elevated expression of hs-CRP. This result implied that KCNQ1OT1 might be related to the activated early inflammatory response in the recurrence of TIA.

Nevertheless, there were still limitations to the research. Multiple-center research studies should be conducted to obtain more subjective data. Neither carotid stenosis nor low-density lipoprotein cholesterol (LDL-C) was identified with significant difference in patients who suffered subsequent stroke. It may be due to the relatively small sample size. More participants are needed in further studies.

In conclusion, patients with upregulated KCNQ1OT1 expression were at a high risk of further ischemic stroke after TIA. Our results indicated that KCNQ1OT1 had great predictive value for further ischemic events, especially when taken together with the ABCD2 score. Patients with the expression level of KCNQ1OT1 over 1.29 had a robust increased risk for further ischemic events. It would be due to the early inflammatory response after TIA, which was reflected by the hs-CRP level. These findings revealed the crucial roles of KCNQ1OT1 in ischemic events in patients with TIA. The underlying relationship between KCNQ1OT1 and early inflammation was discussed for the first time. However, there were still some gaps left in our study. The number of participants was relatively limited. More patients could be recruited to support our conclusion. Molecular biology experiments are needed to explore more specific regulatory mechanisms in our further study.

## Data Availability

The raw data supporting the conclusion of this article will be made available by the authors, without undue reservation.
